# Persistence of Hepatitis B Virus Infection: A Multi-Faceted Player for Hepatocarcinogenesis

**DOI:** 10.3389/fmicb.2021.678537

**Published:** 2021-08-30

**Authors:** Suchandrima Ghosh, Anannya Chakraborty, Soma Banerjee

**Affiliations:** Centre for Liver Research, School of Digestive and Liver Diseases, Institute of Post Graduate Medical Education and Research, Kolkata, India

**Keywords:** hepatitis B Virus, HBV, HBV persistence, hepatocellular carcinoma, HCC

## Abstract

Hepatitis B virus (HBV) infection has a multi-dimensional effect on the host, which not only alters the dynamics of immune response but also persists in the hepatocytes to predispose oncogenic factors. The virus exists in multiple forms of which the nuclear localized covalently closed circular DNA (cccDNA) is the most stable and the primary reason for viral persistence even after clearance of surface antigen and viral DNA. The second reason is the existence of pregenomic RNA (pgRNA) containing virion particles. On the other hand, the integration of the viral genome in the host chromosome also leads to persistent production of viral proteins along with the chromosomal instabilities. The interferon treatment or administration of nucleot(s)ide analogs leads to reduction in the viral DNA load, but the pgRNA and surface antigen clearance are a slow process and complete loss of serological HBsAg is rare. The prolonged exposure of immune cells to the viral antigens, particularly HBs antigen, in the blood circulation results in T-cell exhaustion, which disrupts immune clearance of the virus and virus-infected cells. In addition, it predisposes immune-tolerant microenvironment, which facilitates the tumor progression. Thus cccDNA, pgRNA, and HBsAg along with the viral DNA could be the therapeutic targets in the early disease stages that may improve the quality of life of chronic hepatitis B patients by impeding the progression of the disease toward hepatocellular carcinoma.

## Introduction

**FIGURE 1:**
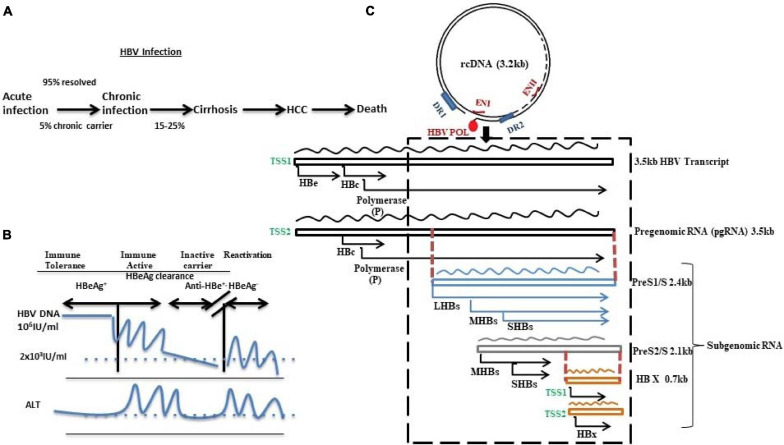
Schematic diagram representing **(A)** HBV infection and disease progression, **(B)** HBV DNA and ALT level in different disease phases, and **(C)** HBV genome as 3.2 kb relaxed circular DNA (rcDNA) with 5′-covalently bound HBV polymerase possessing direct repeat 1 (DR1) and direct repeat 2 (DR2) along with 2 enhancers ENI and ENII. The HBV genome transcribes to generate different sets of RNAs: pregenomic RNA (3.5 kb) and subgenomic RNAs like PreS1/S (2.4 kb), PreS2/S (2.1 kb), and HBX (0.7 kb). The RNAs contain overlapping (denoted by dotted red lines) and frame-shifted ORFs (depicted by the AUG start codon) for each of the viral proteins. The dotted box indicates the most variable region and mutation hotspot of HBV.

Despite being a vaccine-preventable viral infection, hepatitis B virus (HBV) infection is a major global health problem. It is transmitted through perinatal, percutaneous, and sexual exposures. This virus replicates in the hepatocytes of the liver, and in 90% of adults, it manifests self-limiting acute infection with the elimination of the viral DNA and development of anti-core antibody, and seroconversion of HBe-antigen and HBs-antigen (Liang, 2009). In contrast, persistence of HBs-antigen for more than 6 months in adults or perinatal transmission in case of newborn babies in the absence of immunoprophylaxis develops chronic infection (Trehanpati et al., 2013). The natural history of chronic hepatitis B (CHB) phases comprise (1) HBe-Ag^+^ phase that includes immune tolerant phase (no hepatitis), and immune active, or immune clearance phase (active hepatitis phase), and (2) anti-HBe^+^/HBe-Ag^–^ phase including inactive carrier (minimum or no hepatitis) and reactivation (HBe-Ag^–^ active hepatitis) (Figure 1). Thus, CHB (both HBeAg^+^ and HBeAg^–^) often causes life-threatening end-stage liver diseases such as liver cirrhosis (LC) and hepatocellular carcinoma (HCC). Although the infection rate has dropped in children under 5 years of age to 1% because of the global vaccination program, a WHO report suggests that the estimated number of HBV chronic carriers is about 257 million. The most astonishing data is that as of 2015, deaths from this virus mainly related to LC and HCC and it almost reached 8.8 million (WHO, 2020).

The uniqueness in HBV genome organization and its complex life cycle have a multi-dimensional effect on the virus–host interaction, and its persistence leads to predisposition of oncogenic factors. Multiple aspects in HBV infection are related to both viral persistence and disease progression such as (1) the complex life cycle of this partially double-stranded DNA virus mediated through myriads of RNA intermediates and its presence as virion particles, (2) viral genome encoded transactivator proteins and their gene regulatory functions in a multifunctional way, (3) integration of the virus genome into the host chromosome causing genome instabilities, (4) accumulation of immune escape mutations in antigenic viral proteins, and (5) virus–host interaction to suppress immune response (Hai et al., 2014; Tan et al., 2015; Hu et al., 2019; Lazarevic et al., 2019). In addition, nucleotide sequence variations in the viral genome itself dissect drug sensitivity of different viral genotypes and subgenotypes. In this review, the perspectives of viral persistence and the disease progression toward HCC have been delineated, particularly HBV RNA intermediates, drug-resistant covalently closed circular DNA (cccDNA), contribution of genotype/subgenotype and mutations, constitutive presence of viral surface and HBx/N-terminally truncated HBx protein from integrated viral DNA, and defective immune responses, to identify the current lacunae which need more light of research.

## Life Cycle of HBV Through RNA Intermediates: A Key to Disease Pathogenesis

### HBV RNAs and Its Origin

Hepatitis B virus is the smallest enveloped partially double-stranded or relaxed circular DNA (rcDNA) virus with genome length of 3.2 kb belonging to the *Hepadnaviridae* family. Its genome encompasses four open reading frames (ORFs) with overlapped sequences: pre-C/C (pre-core/core), pre-S/S (surface proteins), X (transcriptional co-activator), and P (DNA polymerase) (Figure 1). During infection, the virus particles enter into the cytoplasm of the hepatocyte through a non-covalent attachment of the 77aa of the viral surface protein to the glycosaminoglycans such as heparin sulfate followed by internalization through a highly specific binding to the sodium taurocholate co-transporting polypeptide (NTCP) receptor, and decoates the genome (Le Seyec et al., 1998, 1999; Meier and Stieger, 2002; Yan et al., 2012; Somiya et al., 2016). The rcDNA converts into covalently closed circular DNA (cccDNA) using host repair enzymes in the nucleus (Koniger et al., 2014; Cui et al., 2015; Qi et al., 2016; Long et al., 2017; Kitamura et al., 2018; Tang et al., 2019; Xia and Guo, 2020). This cccDNA acts as a template for all the HBV RNAs. Recent studies have showed that the diversified distribution of the transcription start sites (TSSs) in the HBV genome along with their differential engagement with the transcription factors and RNA polymerase result in heterogeneity in the viral RNA pool (Altinel et al., 2016). Canonically, two different TSSs of the core promoter generate two different 3.5-kb transcripts, one having the start codon for pre-core (preC) and the other originating 4 bp downstream of pre-core start codon generating pgRNA. Recently, splice variant of pgRNA has been also reported in CHB patients (Chen et al., 2015; Duriez et al., 2017). A similar heterogeneity is observed in the transcription of surface proteins: (1) preS1 promoter encodes for large protein (LHBs of 389aa/400aa) and (2) preS/S promoters located within the preS1 gene give rise to middle (MHBs of 281aa) and small (SHBs of 226aa) surface proteins, respectively. Despite predominance of the promoter activity of preS/S, TSS usage determines the ratio of MHBs and SHBs. Lastly, 0.7 kb X mRNA encodes HBx protein and an N-terminally truncated HBx protein, the origin of which is the alternate TSS located in between the first and second codons of HBx (Will et al., 1987; Yaginuma et al., 1993; Quarleri, 2014; Altinel et al., 2016).

The uniqueness of pgRNA is that it is not only used as template for translation of viral polymerase (P) and core protein, it is reverse transcribed into viral DNA after packaging in viral capsid with a single unit of polymerase protein and rcDNA is produced within the capsid (Nassal, 1992, 2008; Weber et al., 1994; Jeong et al., 2000). This newly synthesized viral capsid can reinfect the nucleus to replenish the cccDNA pool and the remaining capsids are enveloped in endoplasmic reticulum and then secreted through Golgi bodies in the blood as infectious Dane particle. Thus, pgRNA, spliced pgRNAs, preS2/PreS1/S RNA, and HBx RNA could be the major factors for disease progression toward HCC. Woodchuck hepatitis model with Woodchuck hepatitis virus (WHV) also revealed similar causal factors for liver disease progression (Mason et al., 2005).

### Impact of HBV RNAs on Persistence of CHB

A few recent evidences suggest that intrahepatic and circulatory pgRNAs could be the predictor for the transcriptional activity of the cccDNA in HBV-positive hepatocytes and the level of pgRNA correlates with the natural course of HBV infection (Wang et al., 2018a,b; Lin et al., 2020). For example, pgRNA level is higher in HBe^+^ compared with HBe^–^ patients, and it is lowest in inactive carriers (Liu Y. et al., 2019). Again in treatment naive patients serum HBV RNA or pgRNA correlates well with both intrahepatic cccDNA as well as with serum HBV DNA and HBsAg level. Such correlation between serum HBV RNA, serum HBV DNA and HBsAg is also detectable after treatment (Liu Y. et al., 2019; Lin et al., 2020, Gao et al., 2017). Studies also suggest that pgRNA remains in the serum of the patients who are under nucleot(s)ide analog (NA) therapy for a long time (Butler et al., 2018; Cloherty et al., 2019; Pan et al., 2021) when viral DNA copy number reduces to an undetectable level. The NA therapy does not completely inhibit the reverse transcription from pgRNA and thus there is a risk of viral reactivation after cessation of NA treatment. Although published data suggest that HBV RNA plays an important role in HBV-mediated disease progression directly or indirectly, the role of pgRNA in the secreted virion-like particle and the infection potential of this particle need to be studied further. Thus, pgRNAs may be considered as a new serum biomarker for judging HBV infection, treatment response, and disease prognosis (Li et al., 2013; Wang et al., 2013; Deng et al., 2016; Takata et al., 2016; Halgand et al., 2018; Wang et al., 2018b; Cloherty et al., 2019).

### HBV RNAs in the Development of HCC

Although the impact of HBV RNA in HCC development has been rarely reported, Ding et al. (2021) showed evidences that the high serum pgRNA level is associated with poor HCC survival and higher recurrence level after hepatectomy. It enhances overall cell proliferation, stemness, and tumorigenicity by overexpressing IGF2BP3 oncoprotein (Ding et al., 2021). Hence, pgRNA may be considered as a detector of HCC and also as a therapeutic target. Contrastingly, using 99 tumor tissues, Halgand et al. (2018) have depicted that both pgRNA and cccDNA in tumors are correlated to the absence of microvascular invasion and survival of the patients (Halgand et al., 2018). The gene expression analysis revealed that pgRNA-positive HCC had low expression of cell cycle and DNA repair genes, while higher expression of NTCP receptors and HBV DNA indicates well-differentiated HCC (Halgand et al., 2018; Ding et al., 2021). Thus, quantification of pgRNA from the non-integrated HBV could be a marker for viral replication and disease prognosis. Altinel et al. (2016) have also showed that nontumor (NT) liver tissue has twofold more HBV expression with a predominance of preS2/S mRNA while the expression of preS1, core, and X were lower in the tumor (T) as observed by the transcriptional mapping using CAGE (cap analysis of gene expression) (Altinel et al., 2016).

In contrast to the heterogeneous pool of HBV transcripts detected in the liver, the corresponding blood samples showed predominance in pgRNA. In addition, shorter HBx transcripts encoding N-terminally truncated HBxAg were found in abundance in both T and NT compared with the canonical HBx, which is consistent with the reports suggesting that truncated HBxAg plays an important role in hepatocarcinogenesis (Altinel et al., 2016).

Apart from these canonical transcripts, about 22 spliced variants (spHBV) have also been detected in HBV-infected patients; 18 variants of pgRNA and 4 of preS2/S (Chen et al., 1989, 2015; Su et al., 1989; Terré et al., 1991; Wu et al., 1991; Rosmorduc et al., 1995; Günther et al., 1997; Sommer et al., 2000; Hass et al., 2005; Abraham et al., 2008; Candotti et al., 2012; El Chaar et al., 2012; Betz-Stablein et al., 2016; Lam et al., 2017; Ito et al., 2019). Among these, hepatitis B spliced protein (HBSP), which originates from 2.2-kb splice variants, are abundantly found in both T and NT tissues (Lin et al., 2002; Soussan et al., 2003). Ectopic expression of HBSP in hepatoma cells restricts TNFα-mediated hepatic inflammation and subsequently reduces the immune infiltration in liver. In addition, it can also promote anti-apoptotic effect by inducing PI3K/AKT activity and preventing FAS-induced apoptosis (Pol et al., 2015; Wu et al., 2018). Its interaction with cathepsin B promotes cellular migration and invasion via activating MMP9 and urokinase-type plasminogen activator (uPA), enhancing tumor-induced vascularization of endothelial cells and activation of MAPK/AKT Signaling (Chen et al., 2012). Bayliss et al. (2013) investigated the level of spHBV in a cohort of 58 HBV patients from pre-HCC state to post-HCC state and found that ∼38% patients had high spHBV level 1–3 years before HCC diagnosis while the percentage rose to 50% during the diagnosis of HCC (Bayliss et al., 2013). Thus, the level of spHBV could be a good HCC prognostic marker.

### Impact of cccDNA on Persistence of Liver Diseases

Hepatic nuclear cccDNA serves as a repertoire of HBV DNA in patients. Several evidences have suggested that the virological relapse of hepatitis after completion of NA therapy often occurs because of the presence of nuclear cccDNA, which is not affected by NA as these are inhibitors of reverse transcriptase. Thus, the synthesis of pgRNA and viral proteins is continued for a certain period after therapy. Only a few copies of cccDNA are sufficient to reactivate the HBV replication after therapy withdrawal. Various advanced techniques such as direct cccDNA degradation strategies including Crisper-Cas9 (Ramanan et al., 2015; Yang et al., 2020), transcription activator-like effector nucleases (TALENs) (Chen et al., 2014; Bloom et al., 2019), and zinc finger nucleases (ZFNs) (Cradick et al., 2010) have been investigated. More conclusive studies may be needed to test the efficacy and safety of the molecules that could be used in the clinical trials.

The cccDNA is stable in the quiescent hepatocyte and it acts as a viral mini-chromosome organized by host nuclear histones and non-histone proteins, which protect the DNA from intracellular DNase. The level of cccDNA in the nucleus is independent of HBV-DNA level; treatment of HBV neutralizing antibody could not block cccDNA as observed in cell culture model (Dandri and Peterson, 2020). It is epigenetically regulated because it consists of six CpG islands of which three are conventional and involve in silencing by methylation. DNMTs reduce pgRNA transcription and hence restrict reverse transcription of viral genome (Zhang et al., 2013, 2014; Jain et al., 2015; Zhu et al., 2019). Viral *trans-*activating protein HBx is an important factor in de-silencing cccDNA, which is involved in its hyperacetylation either by blocking the inhibitory effect of methyltransferases like PRMT1 and SETDB1 or Tudor-domain protein Spindlin-1 (Benhenda et al., 2013; Ducroux et al., 2014; Riviere et al., 2015). Depuration by APOBEC3 deaminases in the presence of IFN-α and TNF-α can also reduce cccDNA (Lucifora et al., 2014), but the concentration of IFN-α used for HBV elimination in patients does not affect cccDNA (Niederau et al., 1996; Lau et al., 2005).

On the other hand, the cccDNA is the origin of HBV DNA; the quantification of cccDNA requires liver tissue which is not feasible in all patients. Several ongoing studies have found a correlation between serum pgRNA and transcriptional activity of cccDNA in HBV patients. Gao et al. (2017) have showed that in pretreated condition, HBV-DNA level better reflects intrahepatic cccDNA level in comparison with HBV-RNA and HBsAg, while after 96 weeks of NA therapy, intracellular cccDNA level correlates mainly with HBsAg level (Gao et al., 2017; Huang et al., 2018; Wang et al., 2018b; Liu Y. et al., 2019). Giersch et al. (2017) have also reported that serum pgRNA level reflects the intracellular pgRNA in humanized uPA/SCID/beige mice model of HBV after NA therapy while in NA untreated mice, serum pgRNA level was comparable with both the intracellular pgRNA and cccDNA level (Giersch et al., 2017). Thus, it is important to quantify both serum HBV pgRNA and serum HBV DNA than serum HBV DNA alone before withdrawal of NA therapy.

Several evidences have showed that HBx also represses several miRNAs like miR-138, miR-224, and miR-596, which inhibits viral replication by targeting the HBV pregenomic RNA (pgRNA). HBx also interacts with long non-coding RNA, DLEU2, to relieve the silencing effect of EZH2/PRC2 on the cccDNA transcription. Apart from host liver-specific transcription factors, HBx is also an indispensable factor for an efficient cccDNA transcription (Li et al., 2013; Wang et al., 2013; Deng et al., 2016; Takata et al., 2016; Guerrieri et al., 2017; Salerno et al., 2020).

### Viral Genetic Variability on Persistence of the HBV Infection

Depending on the 8% sequence variations in the entire genome, HBV is genotyped into 10 including A to J, while each genotype is again sub-genotyped on considering 4% sequence alterations (Kramvis, 2014). To date, HBV genotype A (A1–A7), genotype B (B1–B9), genotype C (C1–C6), genotype D (D1–D10), and genotype F (F1–F4) are most studied as these genotypes/subgenotypes showed distinct distribution pattern worldwide along with the disease manifestations. Apart from this, recombinant strains (A/D, A/E, C/D, and G/C) are also observed in different geographical regions (Cui et al., 2002; Simmonds and Midgley, 2005; McMahon, 2009; Araujo, 2015). Recently, C/D recombinant strain of HBV, subgenotype D9, has been found among HBeAg-negative CHB patients from Eastern India (Ghosh et al., 2013). Among these genotypes and subgenotypes, western data showed genotype A–infected patients had high rate of HBV surface antigen clearance along with rapid reduction of viral DNA and sustained improvement of biochemical parameters (Sanchez-Tapias et al., 2002) while a Chinese study depicted that both HBsAg clearance is better for genotypes A and B than genotypes C and D (Lin and Kao, 2015). Studies also suggest that patients with genotypes C and D were more vulnerable to advanced diseases such as liver cirrhosis and HCC (El-Serag, 2012) and showed delayed HBeAg clearance. One study with Alaskan natives reported that genotype C2 and F develop HCC more than genotype A2, B6, and D (McMahon, 2009). In India, subgenotypes D1 and D3 have been found to be associated with advanced diseases though D2 showed highest replication efficiency (Datta et al., 2018; Khatun et al., 2018). Although genotype D has been reported as an independent risk factor for fulminant hepatitis (Thukar et al., 2002; Wai et al., 2005), genotype G is rarely seen alone. It is co-infected with other genotypes particularly genotype A. Genotypes F and H are observed in an indigenous population of South America (Devesa et al., 2004). Patients with genotype C with HBeAg negative are at high risk of progression of CHB to cirrhosis and HCC (Chan et al., 2004).

### Mutations Accumulated in HBV Genome and Its Impact on Disease Progression

Hepatitis B virus, although being a DNA virus, yet exhibits a much higher mutation rate than other DNA viruses (Echevarría and Avellón, 2006). The evolutionary rate of HBV is 10^–5^ to 10^–4^ substitution/site/year, which is almost equivalent to the RNA viruses (Osiowy et al., 2006; Zhou and Holmes, 2007; Wang et al., 2010). Because the ORFs are overlapping in most of the synonymous substitutions in one frame, it may or may not result in synonymous change in the other. For example, deletions in PreS1 and PreS2 or start codon mutations in PreS2 or premature stop codon substitution in S are not only associated with long-lasting chronic HBV infection but are also highly associated with increasing risk for HCC development. High viral load and high replication efficiency result in overload of viral proteins including misfolded and unfolded ones in ER/Golgi apparatus increasing unfolded protein response (UPR) (Lazar et al., 2014; Kim et al., 2017; Choi et al., 2019). Normally, excessive accumulation of envelope proteins in the ER/Golgi apparatus results in budding of dimerized or multimerized envelope proteins without enclosed viral genome leading to secretion of non-infectious spherical or filamentous subviral particles (SVPs) (Patient et al., 2009; Selzer and Zlotnick, 2015). Outnumbered SVPs compared with the infectious virions in the circulation often lead to immune tolerance, which sums up to viral persistence. Moreover, the ratio of L and S envelope proteins is an important deciding factor for a proper assembly and maintenance of cccDNA pool. The differential regulation of the LHBs and other HB proteins is due to the presence of two independent promoters. This leads to a temporal and differential effect of the envelope proteins on the viral life cycle as well as the cellular processes. The promoter for pre-S1 or L envelope proteins is different from pre-S2 or pre-S promoter. Moreover, the production of L proteins is much less than the other surface proteins. Thus, the overproduction of L proteins either due to naturally occurring deletion and/or point mutations in the S promoter CCAAT element or due to differential expression of the host *trans-*acting factors may result in altered viral assembly and secretion. This causes ER stress owing to overload of viral surface proteins. Both the LHBs and the consequent ER stress trigger the synthesis of the MHBs and HBs, which in turn result in increased secretion of both non-infectious and infectious virion particles (Ou and Rutter, 1987; Bock et al., 1999; Summers et al., 1991; Xu et al., 1997; Pollicino et al., 2014). The histological hallmark of chronic HBV infection is ground glass hepatocyte due to excessive accumulation of HBsAg. These hepatocytes possess a huge amount of LHBs containing filamentous particles in the ER/Golgi bodies (Wang et al., 2003; Su et al., 2008).

Mutations in the “a” determinant of HBsAg are very common in chronic HBV patients leaving anti-HBc antibody as the only detectable marker for infection. These mutations lead to alterations in the conformation of the surface antigen offering false-negative result in the commercially available surface antigen detection kit (Weber, 2005; Pawlotsky, 2005). The first vaccine escape mutant identified was glycine to arginine change at 145 residue of HBsAg (sG145R). Selective pressure to persistence of this mutant is observed in case of HBIG administration during liver transplantation (Carman et al., 1990; Ghany et al., 1998). In such event, despite having active HBV infection with high viral load, the detection of HBsAg is limited. Further study with DNA immunization in BALB/C mice revealed that glycosylation of surface could reduce antigenicity (Bolhassani and Yazdi, 2009). Mutations located at T123N, K122I, A159G, and K160N cause over-glycosylation in HBsAg, which also reduces antigenicity and induces viral persistence (Torresi et al., 2002; Wu et al., 2010).

### Integration of HBV DNA and HBV Persistence

Integration of viral DNA into the host chromosome is a very common event observed in CHB and HCC (Mason et al., 2005, 2009, 2010). The parsimonious nature of the viral genome suggests chromosomal integration of the viral genome may not decrease the replication fitness of the virus. Both *in vitro* HBV infection in HepaRG and HepG2 cell line and histological evidences of chronic HBV revealed that HBV integration could be a very early event, and it is considered as one of the reasons for the development of liver cirrhosis and HCC. Woodchuck HBV infection model with respective virus also shows similar event days after infection (Bud and Gerin, 2001; Mason et al., 2005).

The recent next-generation sequencing data suggest that the integration of the viral genome in the non-tumor tissue is dispersed throughout the genome with no hotspot while tumor tissue showed enrichment of particular genomic sites such as MLL4, TERT, and CTNNB1 (Zhao et al., 2016; An et al., 2018). Now the question is about the fate of the integrated DNA. This integrated form cannot produce pgRNA, and it is replication incompetent. The expression of all viral transcripts is altered due to random integration of HBV DNA in the host genome, while only HBsAg transcript expresses constitutively from its native promoter. Other transcripts such as HBeAg/HBcAg may be produced using cellular promoter located upstream of the integration site and the transcript of the oncogenic protein HBx is either truncated or fused with other transcripts (Valenzuela et al., 1979; Edman et al., 1980; Twist et al., 1981; Lee et al., 1999; Podlaha et al., 2019; Zhao et al., 2020).

The frequency of integration of viral DNA is observed more in the tumor tissue (T) than in the non-tumor tissue (NT) (Arbuthnot and Kew, 2001; Hai et al., 2014; Budzinska et al., 2018). Again, intact HBV DNA integration is more common in the chronic hepatitis B and cirrhotic stages than in HCC (Aoki and Robinson, 1989). This could lead to accumulation of deleterious fusion products associated with hepatocarcinogenesis.

The HBV antigens could be expressed from the integrated viral sequences. If only 1% of the hepatocytes express viral antigens and are enriched, then the liver could be a persistent reservoir of HBV proteins. Proteins expressed from the integrated form may support replication of an incompetent strain in *trans*. In addition, accumulation of mutant HBsAg often causes ER stress and UPR, induces stem-like properties, enhances cellular growth, reduces apoptosis, and thus triggers liver disease progression toward HCC.

### Oncogenic Properties of Occult HBV Infection and Persistence of HBV

The occult HBV infection (OBI) in patients with anti-HBsAg is another important reason for the persistence of HBV infection, which causes viral reactivation during immune suppression. Our group has reported recently that among 238 HCV-infected patients, 38 patients (18%) were with OBI while 66.8% were without OBI but having anti-HBs^+^/anti-HBc^+^ (13%), anti-HBs^+^/anti-HBc^–^ (10%), and anti-HBs^–^/anti-HBc^+^ (25%). Here, 90% of patients have common HBsAg-associated mutations in the “a determinant region” T125M and P127T (Mondal et al., 2017). These two mutations were also found among OBI patients in Greek and Egyptian blood donors (Zheng et al., 2004; Mina et al., 2010; Elbahrawy et al., 2015). Using *in vitro* experiment and *in silico* analysis, we have showed that these two mutations do not alter the HBsAg production but antigenicity drops. A similar report was documented by Velay et al. (2016). Hence, these two mutant variants could be the reason of persistent infection by evading the host immunity even in vaccinated hosts. Apart from this, P33S mutation in B-cell epitope located in HBx gene; I42L in PreS2; spH153L and rtS135Y in polymerase; 1050G, A1053G, C1059T, and C1350A in enhancer I and PreS2/S promoter region; and C1637A and T1676A in enhancer II region were found in high number in OBI patients (Mondal et al., 2017). In addition, three mutations in polymerase gene rtV278I, rhL30S, and rhL55I, which generate four alterations in overlapping HBx gene promoter region (G961A, C1249T, T1250C, and C1324A), are also responsible for the low replication efficiency of the virus. In China, PreS1/S mutations T68I and sQ129R/L were found to be associated with anti-HBsAg^+^ OBI, while sS167 and sA166 were found in the major hydrophilic region (Wang et al., 2020). These are all immune escape mutants. Thus, OBI could be the high risk for the development of end-stage diseases.

### Pro-Oncogenic Properties of HBV Proteins on Disease Persistence

Among the viral proteins of HBV, HBx is a multifunctional viral protein having pleiotropic role including development and progression of HCC. Compartmentalized location of HBx within the cell is critical for its various functions in the tumor microenvironment. Presence of this protein in the cytoplasm triggers multiple signal-transduction pathways related to development, invasion, migration, and recurrence of HCC. These include Wnt/β-catenin, nuclear factor κ-light-chain enhancer of activated B cells (NF-κB), Janus kinase/signal transducer and activator of transcription (STAT), and Ras/Raf/mitogen-activated protein kinase (MAPK) pathways. Also, nuclear HBx functions as transactivator of gene regulation for several proto-oncogenes (c-Myc, N-Myc and c-Jun) and activating protein-1 (AP-1), NF-κB, and ATF/CREB (Zhang et al., 2006; Ali et al., 2014; Geng et al., 2015). A recent study shows that this viral protein interacts with HIF-1α and HIF-1α target genes like lysyl oxidase (LOX) family in HCC. Thus, it regulates cell-cycle checkpoints, proliferation, apoptosis, and DNA repair. HBx disrupts mitochondrial stability by downregulating various enzymes and promoting reactive oxygen species (ROS) production and also lipid peroxidation. This causes intracellular damage, metastasis, and resistance to cell death. The impact of this protein on HBV replication triggers endoplasmic reticulum stress. HBx also induces autophagy by PI3/AKT/mTOR pathway (Lee et al., 2004; Park et al., 2007; Fu et al., 2016).

Most interestingly, it plays an important role in promoting apoptotic death in virus-specific CD8^+^ T lymphocytes and reduces the production of IFN-γ, which leads to attenuation of immune response and persistence of the disease. Selective regulation of multiple proinflammatory cytokines including IL-8, IL-18, IL-23, and TNF-α triggers HCC pathogenesis (Wu et al., 2010). Thus, the activity of HBx in the tumor microenvironment is a new therapeutic target for HCC.

Mutated preS2 proteins also confer their oncogenic properties by triggering degradation of p27, hyper-phosphorylating pRb, and promoting cell cycle progression. It is also known to promote overexpression of cyclin A, COX2, and TERT. In addition, 3’ deleted preS/S sequence infers its oncogenic potential by inducing transcription factors like AP1 and NF-κβ and thereby increasing hepatocyte proliferation (Yen et al., 2016). Overall, the persistent HBV life cycle directly confers to the malignant transformation of the hepatocytes.

### Failure of Immune Control and HBV Persistence

In the immune active phase, persistent HBV infection leads to the progression of the underlying chronic liver diseases characterized by persistent inflammation and immune dysfunction (Tan et al., 2015). The pro-inflammatory cytokines like IL-2, IL-7, IL-6, and IFN-γ are overproduced in the milieu leading to necro-inflammation, cellular stress, epigenetic modulations, and DNA damage (Abb et al., 1985; Bozkaya et al., 2000; Lan et al., 2015; Zhong et al., 2016). Such persistent hepatocyte damage and chronic antigen stimulation activate the non-parenchymal cells, alter intracellular cross-talks and T-cell exhaustion leading to tumorigenesis, and develop a tumor immune microenvironment (TIME), which is immunosuppressive in nature. The prime player in maintaining such immunosuppressive microenvironment is the increased abundance of Tregs via HBx-stimulated production of transforming growth factor-β1 (TGF-β1) and increased frequency of monocytic-myeloid derived suppressor cells (mMDSCs) (Li et al., 2016; Pal et al., 2019). mMDSCs and Treg cells may lead to overexpression of IL10, which results in persistent immunosuppressive nature of TIME in HBV-infected HCC (Chen et al., 2011; Kondo and Shimosegawa, 2015; Tu et al., 2016). Blocking of PD-1/PD-L1 axis in chronic HBV patients can partially reverse the immune suppression by reducing the T-cell exhaustion (Féray and López-Labrador, 2019; Li et al., 2020). Moreover, HBV persistence leads to accumulation of surface proteins in the ER and triggers ER stress, which may release exosomal miRNAs like miR-23a-3p that in turn can upregulate PD-L1 expression by tumor-associated macrophages (TAMs) (Liu J. et al., 2019). In addition, exosomes from HBV-infected cells express interferon-induced transmembrane protein 2 (IFITM2), which inhibits the synthesis of IFN-α in clonally expanded plasmacytoid dendritic cells (Shi et al., 2019). Impairment of CD4^+^ follicular helper T cells may also potentiate development of HCC in HBV-positive patients (Chen and Tian, 2019). However, there is an overall immunological heterogeneity in HCC due to difference in infiltrated leukocyte composition in TIME. Leukocyte-enriched HCC is termed as inflamed HCC while poor leukocyte enrichment is known as non-inflamed HCC (Trujillo et al., 2018). In HBV-associated HCC, increased M2 polarization of macrophages mainly mediated by HBsAg stimulation results in enhanced production of anti-inflammatory IL-10 and TGF-β, probably via inhibition of JNK, ERK, and NF-κB signaling (Yang et al., 2014). Thus, failure of immune control is one of the major reasons of HBV persistence and disease progression toward HCC.

## Conclusion

**FIGURE 2:**
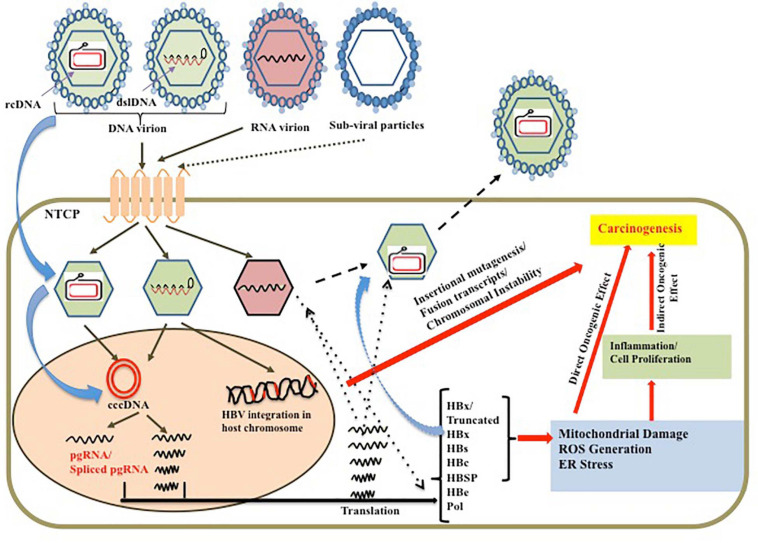
Schematic representation of the causal factors for HBV persistence leading to predisposed oncogenic effect on liver along with the direct oncogenic effect of the viral genome and proteins on hepatocarcinogenesis.

Hepatitis B virus is a unique DNA virus which has a complex strategy to persist inside the cell. It exists as a minichromosome-like structure called cccDNA in the host nucleus, which maintains the viral reservoir and serves as the source of the viral RNA and viral proteins. The complex viral replication strategy leads to generation of myriads of RNA intermediates including the pre-genomic RNA (pgRNA) and other subgenomic RNAs. The specialty of the viral genome is in its overlapping and frame-shifted ORFs, which results in differential regulation and expression of the viral proteins. In addition, such overlapping sequences result in the synergistic effect of the mutations in more than one frame. Such genomic variability along with the overload of the viral proteins in ER/Golgi apparatus increases the UPR thereby leading to stress. Such prolonged induction of stress associated with direct or indirect effect of the virus on the cellular apoptosis and proliferation results in the disease progression toward carcinoma. Moreover, the viral oncoprotein HBx is one of the prime factors that interacts with several host proteins and modulates the signaling pathways to aid in the process of hepatocarcinogenesis. In addition, the integration process not only increases the genome instability but also generates several fused and/or truncated or altered viral and host proteins, which aids in the hepatocarcinogenesis process. Lastly, the prolonged exposure to HBsAg in the circulation results in the imbalance in the immune response leading to development of an immune-tolerant microenvironment, which facilitates the tumor progression. The overview of the complex crosstalk of the viral persistence with hepatocarcinogenesis is presented in Figure 2.

## Author Contributions

SG and AC had prepared the manuscript. SB had thoroughly read and finalized the manuscript. All authors contributed to the article and approved the submitted version.

## Conflict of Interest

The authors declare that the research was conducted in the absence of any commercial or financial relationships that could be construed as a potential conflict of interest.

## Publisher’s Note

All claims expressed in this article are solely those of the authors and do not necessarily represent those of their affiliated organizations, or those of the publisher, the editors and the reviewers. Any product that may be evaluated in this article, or claim that may be made by its manufacturer, is not guaranteed or endorsed by the publisher.

## References

[B1] Abb J. Zachoval R. Eisenburg J. Pape G. R. Zachoval V. Deinhardt F. (1985). Production of interferon alpha and interferon gamma by peripheral blood leukocytes from patients with chronic hepatitis B virus infection. *J. Med. Virol.* 16 171–176. 3925078 10.1002/jmv.1890160209

[B2] Abraham T. M. Lewellyn E. B. Haines K. M. Loeb D. D. (2008). Characterization of the contribution of spliced RNAs of hepatitis B virus to DNA synthesis in transfected cultures of Huh7 and HepG2 cells. *Virology* 379 30–37. 10.1016/j.virol.2008.06.021 18657840 PMC2603046

[B3] Ali A. Abdel-Hafiz H. Suhail M. Al-Mars A. Zakaria M. K. Fatima K. (2014). Hepatitis B virus, HBx mutants and their role in hepatocellular carcinoma. *World J. Gastroenterol.* 20 10238–10248. 10.3748/wjg.v20.i30.10238 25132741 PMC4130832

[B4] Altinel K. Hashimoto K. Wei Y. Neuveut C. Gupta I. Suzuki A. M. (2016). Single-nucleotide resolution mapping of hepatitis B virus promoters in infected human livers and hepatocellular carcinoma. *J. Virol.* 90 10811–10822. 10.1128/jvi.01625-16 27681123 PMC5110153

[B5] An P. Xu J. Yu Y. Winkler C. A. (2018). Host and viral genetic variation in HBV-related hepatocellular carcinoma. *Front. Genet.* 9:261. 10.3389/fgene.2018.00261 30073017 PMC6060371

[B6] Aoki N. Robinson W. S. (1989). State of hepatitis B viral genomes in cirrhotic and hepatocellular carcinoma nodules. *Mol. Biol. Med.* 6 395–408. 2560524

[B7] Araujo N. M. (2015). Hepatitis B virus intergenotypic recombinants worldwide: an overview. *Infect. Genet. Evol.* 36 500–510. 10.1016/j.meegid.2015.08.024 26299884

[B8] Arbuthnot P. Kew M. (2001). Hepatitis B virus and hepatocellular carcinoma. *Int. J. Exp. Pathol.* 82 77–100. 11454100 10.1111/j.1365-2613.2001.iep0082-0077-x PMC2517704

[B9] Bayliss J. Lim L. Thompson A. J. V. Desmond P. Angus P. Locarnini S. (2013). Hepatitis B virus splicing is enhanced prior to development of hepatocellular carcinoma. *J. Hepatol.* 59 1022–1028. 10.1016/j.jhep.2013.06.018 23811301

[B10] Benhenda S. Ducroux A. Rivière L. Sobhian B. Ward M. D. Dion S. (2013). Methyltransferase PRMT1 is a binding partner of HBx and a negative regulator of hepatitis B virus transcription. *J. Virol.* 87 4360–4371. 10.1128/jvi.02574-12 23388725 PMC3624337

[B11] Betz-Stablein B. D. Töpfer A. Littlejohn M. Yuen L. Colledge D. Sozzi V. (2016). Single-molecule sequencing reveals complex genome variation of hepatitis B virus during 15 years of chronic infection following liver transplantation. *J. Virol.* 90 7171–7183. 10.1128/jvi.00243-16 27252524 PMC4984637

[B12] Bloom K. Kaldine H. Cathomen T. Mussolino C. Ely A. Arbuthnot P. (2019). Inhibition of replication of hepatitis B virus using transcriptional repressors that target the viral DNA. *BMC Infect. Dis.* 19:802. 10.1186/s12879-019-4436-y 31510934 PMC6739920

[B13] Bock C. T. Tillmann H. L. Manns M. P. Trautwein C. (1999). The pre-S region determines the intracellular localization and appearance of hepatitis B virus. *Hepatology* 30 517–525. 10.1002/hep.510300206 10421662

[B14] Bolhassani A. Yazdi S. R. (2009). DNA immunization as an efficient strategy for vaccination. *Avicenna J. Med. Biotechnol.* 1 71–88. 23407787 PMC3558129

[B15] Bozkaya H. Bozdayi M. Türkyilmaz R. Sarioglu M. Cetinkaya H. Cinar K. (2000). Circulating IL-2, IL-10 and TNF-alpha in chronic hepatitis B: their relations to HBeAg status and the activity of liver disease. *Hepatogastroenterology* 47 1675–1679. 11149030

[B16] Bud C. T. Gerin J. L. (2001). The woodchuck model of hepatitis B virus infection. *ILAR J.* 42 89–102. 11406711 10.1093/ilar.42.2.89

[B17] Budzinska M. A. Shackel N. A. Urban S. Tu T. (2018). Cellular genomic sites of hepatitis B virus DNA integration. *Genes* 9:365. 10.3390/genes9070365 30037029 PMC6071206

[B18] Butler E. K. Gersch J. McNamara A. Luk K. C. Holzmayer V. de Medina M. (2018). Hepatitis B virus serum DNA and RNA levels in nucleos(t)ide analog-treated or untreated patients during chronic and acute infection. *Hepatology* 68 2106–2117. 10.1002/hep.30082 29734472

[B19] Candotti D. Lin C. K. Belkhiri D. Sakuldamrongpanich T. Biswas S. Lin S. (2012). Occult hepatitis B infection in blood donors from South East Asia: molecular characterisation and potential mechanisms of occurrence. *Gut* 61 1744–1753. 10.1136/gutjnl-2011-301281 22267593

[B20] Carman W. F. Zanetti A. R. Karayiannis P. Waters J. Manzillo G. Tanzi E. (1990). Vaccine-induced escape mutant of hepatitis B virus. *Lancet* 336 325–329. 10.1016/0140-6736(90)91874-a 1697396

[B21] Chan H. L. Hui A. Y. Wong M. L. Tse A. M. Hung L. C. Wong V. W. (2004). Genotype C hepatitis B virus infection is associated with an increased risk of hepatocellular carcinoma. *Gut* 53 1494–1498. 10.1136/gut.2003.033324 15361502 PMC1774221

[B22] Chen J. Wu M. Wang F. Zhang W. Yuan Z. (2015). Hepatitis B virus spliced variants are associated with an impaired response to interferon therapy. *Sci. Rep.* 5:16459. 26585041 10.1038/srep16459 PMC4653653

[B23] Chen J. Zhang W. Lin J. Wang F. Wu M. Chen C. (2014). An efficient antiviral strategy for targeting hepatitis B virus genome using transcription activator-like effector nucleases. *Mol. Ther.* 22 303–311. 10.1038/mt.2013.212 24025750 PMC3916035

[B24] Chen P. J. Chen C. R. Sung J. L. Chen D. S. (1989). Identification of a doubly spliced viral transcript joining the separated domains for putative protease and reverse transcriptase of hepatitis B virus. *J. Virol.* 63 4165–4171. 10.1128/jvi.63.10.4165-4171.1989 2476567 PMC251030

[B25] Chen S. Akbar S. M. Abe M. Hiasa Y. Onji M. (2011). Immunosuppressive functions of hepatic myeloid-derived suppressor cells of normal mice and in a murine model of chronic hepatitis B virus. *Clin. Exp. Immunol.* 166 134–142. 21762128 10.1111/j.1365-2249.2011.04445.x PMC3193928

[B26] Chen W. N. Chen J. Y. Jiao B. Y. Lin W. S. Wu Y. L. Liu L. L. (2012). Interaction of the hepatitis B spliced protein with cathepsin B promotes hepatoma cell migration and invasion. *J. Virol.* 86 13533–13541. 10.1128/jvi.02095-12 23035214 PMC3503111

[B27] Chen Y. Tian Z. (2019). HBV-induced immune imbalance in the development of HCC. *Front. Immunol.* 10:2048. 31507621 10.3389/fimmu.2019.02048 PMC6718466

[B28] Choi Y. M. Lee S. Y. Kim B. J. (2019). Naturally occurring hepatitis B virus mutations leading to endoplasmic reticulum stress and their contribution to the progression of hepatocellular carcinoma. *Int. J. Mol. Sci.* 20:597. 10.3390/ijms20030597 30704071 PMC6387469

[B29] Cloherty G. Butler E. Kuhns M. (2019). Serum hepatitis B Virus RNA as a potential diagnostic biomarker during chronic hepatitis B virus infection. *Clin. Liver Dis.* 13 90–92. 10.1002/cld.774 30988945 PMC6446447

[B30] Cradick T. J. Keck K. Bradshaw S. Jamieson A. C. McCaffrey A. P. (2010). Zinc-finger nucleases as a novel therapeutic strategy for targeting hepatitis B virus DNAs. *Mol. Ther.* 18 947–954. 10.1038/mt.2010.20 20160705 PMC2890117

[B31] Cui C. Shi J. Hui L. Xi H. Zhuoma Quni. (2002). The dominant hepatitis B virus genotype identified in Tibet is a C/D hybrid. *J. Gen. Virol.* 83 2773–2777. 10.1099/0022-1317-83-11-2773 12388813

[B32] Cui X. McAllister R. Boregowda R. Sohn J. A. Cortes Ledesma F. Caldecott K. W. (2015). Does tyrosyl DNA phosphodiesterase-2 play a role in hepatitis B virus genome repair? *PLoS One* 10:e0128401. 10.1371/journal.pone.0128401 26079492 PMC4469307

[B33] Dandri M. Peterson J. (2020). cccDNA maintenance in chronic hepatits B-Targeting the matrix of viral replication. *Infect. Drug Resist.* 2020 3873–3886. 10.2147/IDR.S240472 PMC7605611 33149632

[B34] Datta S. Dasgupta D. Ghosh A. Ghosh S. Manna A. Datta S. (2018). Oncogenic potential of hepatitis B virus subgenotype D1 surpasses D3: significance in the development of hepatocellular carcinoma. *Carcinogenesis* 39 283–292. 10.1093/carcin/bgx145 29228221

[B35] Deng M. Hou J. Hu J. Wang S. Chen M. Chen L. (2016). Hepatitis B virus mRNAs functionally sequester let-7a and enhance hepatocellular carcinoma. *Cancer Lett.* 383 62–72. 10.1016/j.canlet.2016.09.028 27693636

[B36] Devesa M. Rodriguez C. Leon G. Liprandi F. Pujol F. H. (2004). Clade analysis and surface antigen polymorphism of hepatitis B virus American genotypes. *J. Med. Virol.* 72 377–384. 10.1002/jmv.20015 14748061

[B37] Ding W. B. Wang M. C. Yu J. Huang G. Sun D. P. Lei L. (2021). HBV-pgRNA increases the stemness and promotes the development of HBV-related HCC through reciprocal regulation with IGF2BP3. *Hepatology* 7. 10.1002/hep.31850 33825218

[B38] Ducroux A. Benhenda S. Riviere L. Semmes O. J. Benkirane M. Neuveut C. (2014). The Tudor domain protein Spindlin1 is involved in intrinsic antiviral defense against incoming hepatitis B Virus and herpes simplex virus type 1. *PLoS pathogens* 10:e1004343. 10.1371/journal.ppat.1004343 25211330 PMC4161474

[B39] Duriez M. Mandouri Y. Lekbaby B. Wang H. Schnuriger A. Redelsperger F. (2017). Alternative splicing of hepatitis B virus: a novel virus/host interaction altering liver immunity. *J. Hepatol.* 67 687–699. 10.1016/j.jhep.2017.05.025 28600137 PMC6433284

[B40] Echevarría J. M. Avellón A. (2006). Hepatitis B virus genetic diversity. *J. Med. Virol.* 78(Suppl.1) S36–S42. 16622876 10.1002/jmv.20605

[B41] Edman J. C. Gray P. Valenzuela P. Rall L. B. Rutter W. J. (1980). Integration of hepatitis B virus sequences and their expression in a human hepatoma cell. *Nature* 286 535–538. 6250075 10.1038/286535a0

[B42] Elbahrawy A. Alaboudy A. El Moghazy W. Elwassief A. Alashker A. Abdallah A. M. (2015). Occult hepatitis B virus infection in Egypt. *World J. Hepatol.* 7 1671–1678. 10.4254/wjh.v7.i12.1671 26140086 PMC4483548

[B43] El Chaar M. El Jisr T. Allain J.-P. (2012). Hepatitis B virus DNA splicing in Lebanese blood donors and genotype A to E strains: implications for hepatitis B virus DNA quantification and infectivity. *J. Clin. Microbiol.* 50 3159–3167. 10.1128/jcm.01251-12 22785194 PMC3457458

[B44] El-Serag H. B. (2012). Epidemiology of viral hepatitis and hepatocellular carcinoma. *Gastroenterology* 142 1264.e–1273.e. 22537432 10.1053/j.gastro.2011.12.061 PMC3338949

[B45] Féray C. López-Labrador F. X. (2019). Is PD-1 blockade a potential therapy for HBV? *JHEP Rep.* 1 142–144. 32040093 10.1016/j.jhepr.2019.07.007 PMC7001582

[B46] Fu S. Zhou R. R. Li N. Huang Y. Fan X. G. (2016). Hepatitis B virus X protein in liver tumor microenvironment. *Tumour Biol.* 37 15371–15381. 10.1007/s13277-016-5406-2 27658781 PMC5250643

[B47] Gao Y. Li Y. Meng Q. Zhang Z. Zhao P. Shang Q. (2017). Serum hepatitis B virus DNA, RNA, and HBsAg: which correlated better with intrahepatic covalently closed circular DNA before and after nucleos(t)ide analogue treatment? *J. Clin. Microbiol.* 55 2972–2982. 10.1128/jcm.00760-17 28747369 PMC5625383

[B48] Geng M. Xin X. Bi L. Q. Zhou L. T. Liu X. H. (2015). Molecular mechanism of hepatitis B virus X protein function in hepatocarcinogenesis. *World J. Gastroenterol.* 21 10732–10738. 10.3748/wjg.v21.i38.10732 26478665 PMC4600575

[B49] Ghany M. G. Ayola B. Villamil F. G. Gish R. G. Rojter S. Vierling J. M. (1998). Hepatitis B virus S mutants in liver transplant recipients who were reinfected despite hepatitis B immune globulin prophylaxis. *Hepatology* 27 213–222. 10.1002/hep.510270133 9425940

[B50] Ghosh S. Banerjee P. Deny P. Mondal R. K. Nandi M. Roychoudhury A. (2013). New HBV subgenotype D9, a novel D/C recombinant, identified in patients with chronic HBeAg-negative infection in Eastern India. *J. Viral. Hepat.* 20 209–218. 10.1111/j.1365-2893.2012.01655.x 23383660

[B51] Giersch K. Allweiss L. Volz T. Dandri M. Lutgehetmann M. (2017). Serum HBV pgRNA as a clinical marker for cccDNA activity. *J. Hepatol.* 66 460–462. 10.1016/j.jhep.2016.09.028 27826059

[B52] Guerrieri F. Belloni L. D’Andrea D. Pediconi N. Le Pera L. Testoni B. (2017). Genome-wide identification of direct HBx genomic targets. *BMC Genomics* 18:184. 10.1186/s12864-017-3561-5 28212627 PMC5316204

[B53] Günther S. Sommer G. Iwanska A. Will H. (1997). Heterogeneity and common features of defective hepatitis B virus genomes derived from spliced pregenomic RNA. *Virology* 238 363–371. 10.1006/viro.1997.8863 9400609

[B54] Hai H. Tamori A. Kawada N. (2014). Role of hepatitis B virus DNA integration in human hepatocarcinogenesis. *World J. Gastroenterol.* 20 6236–6243. 10.3748/wjg.v20.i20.6236 24876744 PMC4033461

[B55] Halgand B. Desterke C. Rivière L. Fallot G. Sebagh M. Calderaro J. (2018). Hepatitis B virus pregenomic RNA in hepatocellular carcinoma: a nosological and prognostic determinant. *Hepatology* 67 86–96. 10.1002/hep.29463 28802063

[B56] Hass M. Hannoun C. Kalinina T. Sommer G. Manegold C. Günther S. (2005). Functional analysis of hepatitis B virus reactivating in hepatitis B surface antigen-negative individuals. *Hepatology* 42 93–103. 10.1002/hep.20748 15962285

[B57] Hu J. Protzer U. Siddiqui A. (2019). Revisiting hepatitis B virus: challenges of curative therapies. *J. Virol.* 93:e01032-19. 10.1128/JVI.01032-19 31375584 PMC6798116

[B58] Huang H. Wang J. Li W. Chen R. Chen X. Zhang F. (2018). Serum HBV DNA plus RNA shows superiority in reflecting the activity of intrahepatic cccDNA in treatment-naive HBV-infected individuals. *J. Clin. Virol.* 99–100 71–78. 10.1016/j.jcv.2017.12.016 29353073

[B59] Ito N. Nakashima K. Sun S. Ito M. Suzuki T. (2019). Cell type diversity in hepatitis B virus RNA splicing and its regulation. *Front. Microbiol.* 10:207. 10.3389/fmicb.2019.00207 30800119 PMC6375855

[B60] Jain S. Chang T.-T. Chen S. Boldbaatar B. Clemens A. Lin S. Y. (2015). Comprehensive DNA methylation analysis of hepatitis B virus genome in infected liver tissues. *Sci. Rep.* 5:10478. 26000761 10.1038/srep10478 PMC4650678

[B61] Jeong J. K. Yoon G. S. Ryu W. S. (2000). Evidence that the 5’-end cap structure is essential for encapsidation of hepatitis B virus pregenomic RNA. *J. Virol.* 74 5502–5508. 10.1128/jvi.74.12.5502-5508.2000 10823855 PMC112035

[B62] Khatun M. Mondal R. K. Pal S. Baidya A. Bishnu D. Banerjee P. (2018). Distinctiveness in virological features and pathogenic potentials of subgenotypes D1, D2, D3 and D5 of Hepatitis B virus. *Sci. Rep.* 8:8055. 29795338 10.1038/s41598-018-26414-4 PMC5966457

[B63] Kim S. Y. Kyaw Y. Y. Cheong J. (2017). Functional interaction of endoplasmic reticulum stress and hepatitis B virus in the pathogenesis of liver diseases. *World J. Gastroenterol.* 23 7657–7665. 10.3748/wjg.v23.i43.7657 29209107 PMC5703926

[B64] Kitamura K. Que L. Shimadu M. Koura M. Ishihara Y. Wakae K. (2018). Flap endonuclease 1 is involved in cccDNA formation in the hepatitis B virus. *PLoS Pathog* 14:e1007124. 10.1371/journal.ppat.1007124 29928064 PMC6013022

[B65] Kondo Y. Shimosegawa T. (2015). Significant roles of regulatory T cells and myeloid derived suppressor cells in hepatitis B virus persistent infection and hepatitis B virus-related HCCs. *Int. J. Mol. Sci.* 16 3307–3322. 25654227 10.3390/ijms16023307 PMC4346897

[B66] Koniger C. Wingert I. Marsmann M. Rosler C. Beck J. Nassal M. (2014). Involvement of the host DNA-repair enzyme TDP2 in formation of the covalently closed circular DNA persistence reservoir of hepatitis B viruses. *Proc. Natl. Acad. Sci. U.S.A.* 111 E4244–E4253. 25201958 10.1073/pnas.1409986111 PMC4209993

[B67] Kramvis A. (2014). Genotypes and genetic variability of hepatitis B virus. *Intervirology* 57 141–150. 10.1159/000360947 25034481

[B68] Lam A. M. Ren S. Espiritu C. Kelly M. Lau V. Zheng L. (2017). Hepatitis B virus capsid assembly modulators, but not nucleoside analogs, inhibit the production of extracellular pregenomic RNA and spliced RNA variants. *Antimicrob. Agents Chemother.* 61 e680-17. 10.1128/AAC.00680-17 PMC5527605 28559265

[B69] Lan T. Chang L. Wu L. Yuan Y. F. (2015). IL-6 plays a crucial role in HBV infection. *J. Clin. Transl. Hepatol.* 3 271–276. 26807383 10.14218/JCTH.2015.00024 PMC4721895

[B70] Lau G. K. Piratvisuth T. Luo K. X. Marcellin P. Thongsawat S. Cooksley G. (2005). Peginterferon Alfa-2a, lamivudine, and the combination for HBeAg-positive chronic hepatitis B. *N. Engl. J. Med.* 352 2682–2695. 15987917 10.1056/NEJMoa043470

[B71] Lazar C. Uta M. Branza-Nichita N. (2014). Modulation of the unfolded protein response by the human hepatitis B virus. *Front. Microbiol.* 5:433. 10.3389/fmicb.2014.00433 25191311 PMC4137222

[B72] Lazarevic I. Banko A. Miljanovic D. Cupic M. (2019). Immune-escape hepatitis B virus mutations associated with viral reactivation upon immunosuppression. *Viruses* 11:778. 10.3390/v11090778 31450544 PMC6784188

[B73] Le Seyec J. Chouteau P. Cannie I. Guguen-Guillouzo C. Gripon P. (1998). Role of the pre-S2 domain of the large envelope protein in hepatitis B virus assembly and infectivity. *J. Virol.* 72 5573–5578. 10.1128/jvi.72.7.5573-5578.1998 9621015 PMC110210

[B74] Le Seyec J. Chouteau P. Cannie I. Guguen-Guillouzo C. Gripon P. (1999). Infection process of the hepatitis B virus depends on the presence of a defined sequence in the pre-S1 domain. *J. Virol.* 73 2052–2057. 10.1128/jvi.73.3.2052-2057.1999 9971786 PMC104448

[B75] Lee J. H. Ku J. L. Park Y. J. Lee K. U. Kim W. H. Park J. G. (1999). Establishment and characterization of four human hepatocellular carcinoma cell lines containing hepatitis B virus DNA. *World J. Gastroenterol.* 5 289–295. 10.3748/wjg.v5.i4.289 11819450 PMC4695537

[B76] Lee Y. I. Hwang J. M. Im J. H. Lee Y. I. Kim N. S. Kim D. G. (2004). Human hepatitis B virus-X protein alters mitochondrial function and physiology in human liver cells. *J. Biol. Chem.* 279 15460–15471. 10.1074/jbc.m309280200 14724286

[B77] Li B. Yan C. Zhu J. Chen X. Fu Q. Zhang H. (2020). Anti-PD-1/PD-L1 blockade immunotherapy employed in treating hepatitis B virus infection-related advanced hepatocellular carcinoma: a literature review. *Front. Immunol.* 11:1037. 32547550 10.3389/fimmu.2020.01037 PMC7270402

[B78] Li C. Wang Y. Wang S. Wu B. Hao J. Fan H. (2013). Hepatitis B virus mRNA-mediated miR-122 inhibition upregulates PTTG1-binding protein, which promotes hepatocellular carcinoma tumor growth and cell invasion. *J. Virol.* 87 2193–2205. 10.1128/jvi.02831-12 23221562 PMC3571498

[B79] Li W. Han J. Wu H. (2016). Regulatory T-cells promote hepatitis B virus infection and hepatocellular carcinoma progression. *Chronic Dis. Transl. Med.* 2 67–80. 29063027 10.1016/j.cdtm.2016.09.001 PMC5643754

[B80] Liang T. J. (2009). Hepatitis B: the virus and disease. *Hepatology* 49(5 Suppl.) S13–S21. 19399811 10.1002/hep.22881 PMC2809016

[B81] Lin C. L. Kao J. H. (2015). Hepatitis B virus genotypes and variants. *Cold Spring Harb. Perspect. Med.* 5:a021436. 25934462 10.1101/cshperspect.a021436 PMC4448583

[B82] Lin N. Ye A. Lin J. Liu C. Huang J. Fu Y. (2020). Diagnostic value of detection of pregenomic RNA in sera of hepatitis B virus-infected patients with different clinical outcomes. *J. Clin. Microbiol.* 58 e1275–e1219. 10.1128/JCM.01275-19 PMC6989074 31723011

[B83] Lin X. Wen Y. Wan D. Qian G. Gu J. (2002). Structural and functional analysis of 2.2 kb spliced variant of hepatitis B virus genomes isolated from liver tissues from hepatocellular carcinoma patients. *Zhonghua Shi YanHe Lin Chuang Bing Du Xue Za Zhi* 16 11–15. 11986736

[B84] Liu J. Fan L. Yu H. Zhang J. He Y. Feng D. (2019). Endoplasmic reticulum stress causes liver cancer cells to release exosomal miR-23a-3p and Up-regulate programmed death ligand 1 expression in macrophages. *Hepatology* 70 241–258. 30854665 10.1002/hep.30607 PMC6597282

[B85] Liu Y. Jiang M. Xue J. Yan H. Liang X. (2019). Serum HBV RNA quantification: useful for monitoring natural history of chronic hepatitis B infection. *BMC Gastroenterol* 19:53. 10.1186/s12876-019-0966-4 30991954 PMC6469196

[B86] Long Q. Yan R. Hu J. Cai D. Mitra B. Kim E. S. (2017). The role of host DNA ligases in hepadnavirus covalently closed circular DNA formation. *PLoS Pathog.* 13:e1006784. 10.1371/journal.ppat.1006784 29287110 PMC5747486

[B87] Lucifora J. Xia Y. Reisinger F. Zhang K. Stadler D. Cheng X. (2014). Specific and nonhepatotoxic degradation of nuclear hepatitis B virus cccDNA. *Science* 343 1221–1228. 10.1126/science.1243462 24557838 PMC6309542

[B88] Mason W. S. Jilbert A. R. Summers J. (2005). Clonal expansion of hepatocytes during chronic woodchuck hepatitis virus infection. *Proc. Natl. Acad. Sci. U.S.A.* 102 1139–1144. 10.1073/pnas.0409332102 15657132 PMC544623

[B89] Mason W. S. Liu C. Aldrich C. E. Litwin S. Yeh M. M. (2010). Clonal expansion of normal-appearing human hepatocytes during chronic hepatitis B virus infection. *J. Virol.* 84 8308–8315. 10.1128/jvi.00833-10 20519397 PMC2916518

[B90] Mason W. S. Low H. C. Xu C. Aldrich C. E. Scougall C. A. Grosse A. (2009). Detection of clonally expanded hepatocytes in chimpanzees with chronic hepatitis B virus infection. *J. Virol.* 83 8396–8408. 10.1128/jvi.00700-09 19535448 PMC2738144

[B91] McMahon B. J. (2009). The influence of hepatitis B virus genotype and subgenotype on the natural history of chronic hepatitis B. *Hepatol. Int.* 3 334–342. 10.1007/s12072-008-9112-z 19669359 PMC2716762

[B92] Meier P. J. Stieger B. (2002). Bile salt transporters. *Annu. Rev. Physiol.* 64 635–661. 10.1146/annurev.physiol.64.082201.100300 11826283

[B93] Mina P. Georgiadou S. P. Rizos C. Dalekos G. N. Rigopoulou E. I. (2010). Prevalence of occult hepatitis B virus infection in haemodialysis patients from central Greece. *World J. Gastroenterol.* 16 225–231. 20066742 10.3748/wjg.v16.i2.225 PMC2806561

[B94] Mondal R. K. Khatun M. Banerjee P. Ghosh A. Sarkar S. Santra A. (2017). Synergistic impact of mutations in hepatitis B virus genome contribute to its occult phenotype in chronic hepatitis C virus carriers. *Sci. Rep.* 7:9653. 28852072 10.1038/s41598-017-09965-w PMC5574988

[B95] Nassal M. (1992). The arginine-rich domain of the hepatitis B virus core protein is required for pregenome encapsidation and productive viral positive-strand DNA synthesis but not for virus assembly. *J. Virol.* 66 4107–4116. 10.1128/jvi.66.7.4107-4116.1992 1602535 PMC241213

[B96] Nassal M. (2008). Hepatitis B viruses: reverse transcription a different way. *Virus Res.* 134 235–249. 10.1016/j.virusres.2007.12.024 18339439

[B97] Niederau C. Heintges T. Lange S. Goldmann G. Niederau C. M. Mohr L. (1996). Long-term follow-up of HBeAg-positive patients treated with interferon alfa for chronic hepatitis B. *N. Engl. J. Med.* 334 1422–1427. 10.1056/nejm199605303342202 8618580

[B98] Osiowy C. Giles E. Tanaka Y. Mizokami M. Minuk G. Y. (2006). Molecular evolution of hepatitis B virus over 25 years. *J. Virol.* 80 10307–10314. 10.1128/jvi.00996-06 17041211 PMC1641782

[B99] Ou J. H. Rutter W. J. (1987). Regulation of secretion of the hepatitis B virus major surface antigen by the preS-1 protein. *J. Virol.* 61 782–786. 10.1128/jvi.61.3.782-786.1987 3806798 PMC254020

[B100] Pal S. Nandi M. Dey D. Chakraborty B. C. Shil A. Ghosh S. (2019). Myeloid-derived suppressor cells induce regulatory T cells in chronically HBV infected patients with high levels of hepatitis B surface antigen and persist after antiviral therapy. *Aliment Pharmacol. Ther.* 49 1346–1359. 30982998 10.1111/apt.15226

[B101] Pan J. Xu J. Luo H. Tan N. Kang Q. Chen H. (2021). Factors and virological significance of hepatitis B virus pregenomic RNA status after 5 years of antiviral therapy. *Int. J. Infect. Dis.* 105 418–423. 10.1016/j.ijid.2021.02.116 33676002

[B102] Park N. H. Song I. H. Chung Y. H. (2007). Molecular pathogenesis of hepatitis-B-virus-associated hepatocellular carcinoma. *Gut Liver* 1 101–117. 10.5009/gnl.2007.1.2.101 20485626 PMC2871634

[B103] Patient R. Hourioux C. Roingeard P. (2009). Morphogenesis of hepatitis B virus and its subviral envelope particles. *Cell Microbiol.* 11 1561–1570. 10.1111/j.1462-5822.2009.01363.x 19673892 PMC2909707

[B104] Pawlotsky J. M. (2005). The concept of hepatitis B virus mutant escape. *J. Clin. Virol.* 34 S125–S129. 16461211 10.1016/s1386-6532(05)80021-6

[B105] Podlaha O. Wu G. Downie B. Ramamurthy R. Gaggar A. Subramanian M. (2019). Genomic modeling of hepatitis B virus integration frequency in the human genome. *PLoS One* 14:e0220376. 10.1371/journal.pone.0220376 31356634 PMC6663024

[B106] Pol J. G. Lekbaby B. Redelsperger F. Klamer S. Mandouri Y. Ahodantin J. (2015). Alternative splicing-regulated protein of hepatitis B virus hacks the TNF-alpha-stimulated signaling pathways and limits the extent of liver inflammation. *FASEB J.* 29 1879–1889. 10.1096/fj.14-258715 25630972

[B107] Pollicino T. Cacciola I. Saffioti F. Raimondo G. (2014). Hepatitis B virus PreS/S gene variants: pathobiology and clinical implications. *J. Hepatol.* 61 408–417. 10.1016/j.jhep.2014.04.041 24801416

[B108] Qi Y. Gao Z. Xu G. Peng B. Liu C. Yan H. (2016). DNA polymerase kappa is a key cellular factor for the formation of covalently closed circular DNA of hepatitis B virus. *PLoS Pathog* 12:e1005893. 10.1371/journal.ppat.1005893 27783675 PMC5081172

[B109] Quarleri J. (2014). Core promoter: a critical region where the hepatitis B virus makes decisions. *World J. Gastroenterol.* 20 425–435. 10.3748/wjg.v20.i2.425 24574711 PMC3923017

[B110] Ramanan V. Shlomai A. Cox D. B. Schwartz R. E. Michailidis E. Bhatta A. (2015). CRISPR/Cas9 cleavage of viral DNA efficiently suppresses hepatitis B virus. *Sci. Rep.* 5:10833. 26035283 10.1038/srep10833 PMC4649911

[B111] Riviere L. Gerossier L. Ducroux A. Dion S. Deng Q. Michel M. L. (2015). HBx relieves chromatin-mediated transcriptional repression of hepatitis B viral cccDNA involving SETDB1 histone methyltransferase. *J. Hepatol.* 63:1093-102. 26143443 10.1016/j.jhep.2015.06.023

[B112] Rosmorduc O. Petit M. A. Pol S. Capel F. Bortolotti F. Berthelot P. (1995). *In vivo* and *in vitro* expression of defective hepatitis B virus particles generated by spliced hepatitis B virus RNA. *Hepatology* 22 10–19. 10.1016/0270-9139(95)90346-1 7601398

[B113] Salerno D. Chiodo L. Alfano V. Floriot O. Cottone G. Paturel A. (2020). Hepatitis B protein HBx binds the DLEU2 lncRNA to sustain cccDNA and host cancer-related gene transcription. *Gut* 69 2016–2024. 32114505 10.1136/gutjnl-2019-319637 PMC7569396

[B114] Sanchez-Tapias J. M. Costa J. Mas A. Bruguera M. Rodés J. (2002). Influence of hepatitis B virus genotype on the long-term outcome of chronic hepatitis B in western patients. *Gastroenterology* 123 1848–1856. 10.1053/gast.2002.37041 12454842

[B115] Selzer L. Zlotnick A. (2015). Assembly and Release of Hepatitis B Virus. *Cold Spring Harb. Perspect. Med.* 5:a021394. 10.1101/cshperspect.a021394 26552701 PMC4665036

[B116] Shi Y. Du L. Lv D. Li H. Shang J. Lu J. (2019). Exosomal interferon-induced transmembrane protein 2 transmitted to dendritic cells inhibits interferon alpha pathway activation and blocks anti-hepatitis B virus efficacy of exogenous interferon alpha. *Hepatology* 69 2396–2413. 30723923 10.1002/hep.30548 PMC6593428

[B117] Simmonds P. Midgley S. (2005). Recombination in the genesis and evolution of hepatitis B virus genotypes. *J. Virol.* 79 15467–15476. 10.1128/jvi.79.24.15467-15476.2005 16306618 PMC1316029

[B118] Somiya M. Liu Q. Yoshimoto N. Iijima M. Tatematsu K. Nakai T. (2016). Cellular uptake of hepatitis B virus envelope L particles is independent of sodium taurocholate cotransporting polypeptide, but dependent on heparan sulfate proteoglycan. *Virology* 497 23–32. 10.1016/j.virol.2016.06.024 27420796

[B119] Sommer G. van Bömmel F. Will H. (2000). Genotype-specific synthesis and secretion of spliced hepatitis B virus genomes in hepatoma cells. *Virology* 271 371–381. 10.1006/viro.2000.0331 10860890

[B120] Soussan P. Tuveri R. Nalpas B. Garreau F. Zavala F. Masson A. (2003). The expression of hepatitis B spliced protein (HBSP) encoded by a spliced hepatitis B virus RNA is associated with viral replication and liver fibrosis. *J. Hepatol.* 38 343–348. 10.1016/s0168-8278(02)00422-1 12586301

[B121] Su I. J. Wang H. C. Wu H. C. Huang W. Y. (2008). Ground glass hepatocytes contain pre-S mutants and represent preneoplastic lesions in chronic hepatitis B virus infection. *J. Gastroenterol. Hepatol.* 23 1169–1174. 10.1111/j.1440-1746.2008.05348.x 18505413

[B122] Su T. S. Lai C. J. Huang J. L. Lin L. H. Yauk Y. K. Chang C. M. (1989). Hepatitis B virus transcript produced by RNA splicing. *J. Virol.* 63 4011–4018. 10.1128/jvi.63.9.4011-4018.1989 2760987 PMC250998

[B123] Summers J. Smith P. M. Huang M. J. Yu S. (1991). Morphogenetic and regulatory effects of mutations in the envelope proteins of an avian hepadnavirus. *J. Virol.* 65 1310–1317. 10.1128/jvi.65.3.1310-1317.1991 1995945 PMC239906

[B124] Takata A. Otsuka M. Ohno M. Kishikawa T. Yoshikawa T. Koike K. (2016). Mutual antagonism between hepatitis B viral mRNA and host microRNA let-7. *Sci. Rep.* 6:23237. 26979389 10.1038/srep23237 PMC4793232

[B125] Tan A. Koh S. Bertoletti A. (2015). Immune response in hepatitis B virus infection. *Cold Spring Harb. Perspect. Med.* 5:a021428. 26134480 10.1101/cshperspect.a021428 PMC4526720

[B126] Tang L. Sheraz M. McGrane M. Chang J. Guo J. T. (2019). DNA polymerase alpha is essential for intracellular amplification of hepatitis B virus covalently closed circular DNA. *PLoS Pathog* 15:e1007742. 10.1371/journal.ppat.1007742 31026293 PMC6505960

[B127] Terré S. Petit M. A. Bréchot C. (1991). Defective hepatitis B virus particles are generated by packaging and reverse transcription of spliced viral RNAs *in vivo*. *J. Virol.* 65 5539–5543. 10.1128/jvi.65.10.5539-5543.1991 1895403 PMC249055

[B128] Thukar V. Guptan R. C. Kazim S. N. Malhotra V. Sarin S. K. (2002). Profile, spectrum and significance of HBV genotypes in chronic liver disease patients in the Indian subcontinent. *J. Gastroenterol. Hepatol.* 17 165–170. 10.1046/j.1440-1746.2002.02605.x 11966946

[B129] Torresi J. Earnest-Silveira L. Civitico G. Walters T. E. Lewin S. R. Fyfe J. (2002). Restoration of replication phenotype of lamivudine resistant hepatitis B virus mutants by compensatory changes in the “fingers” subdomain of the viral polymerase selected as a consequence of mutations in the overlapping S gene. *Virology* 299 88–99. 10.1006/viro.2002.1448 12167344

[B130] Trehanpati N. Hissar S. Shrivastav S. Sarin S. K. (2013). Immunological mechanisms of hepatitis B virus persistence in newborns. *Indian J. Med. Res.* 138 700–710. 24434322 PMC3928700

[B131] Tu J. F. Ding Y. H. Ying X. H. Wu F. Z. Zhou X. M. Zhang D. K. (2016). Regulatory T cells, especially ICOS^+^ FOXP3^+^ regulatory T cells, are increased in the hepatocellular carcinoma microenvironment and predict reduced survival. *Sci. Rep.* 6:35056. 27725696 10.1038/srep35056 PMC5057140

[B132] Trujillo J. A. Sweis R. F. Bao R. Luke J. J. (2018). T cell-inflamed versus Non-T cell-inflamed tumors: a conceptual framework for cancer immunotherapy drug development and combination therapy selection. *Cancer Immunol. Res.* 6, 990–1000. 30181337 10.1158/2326-6066.CIR-18-0277 PMC6145135

[B133] Twist E. M. Clark H. F. Aden D. P. Knowles B. B. Plotkin S. A. (1981). Integration pattern of hepatitis B virus DNA sequences in human hepatoma cell lines. *J. Virol.* 37 239–243. 10.1128/jvi.37.1.239-243.1981 6260977 PMC171000

[B134] Valenzuela P. Gray P. Quiroga M. Zaldivar J. Goodman H. M. Rutter W. J. (1979). Nucleotide sequence of the gene coding for the major protein of hepatitis B virus surface antigen. *Nature* 280 815–819. 10.1038/280815a0 471053

[B135] Velay A. Jeulin H. Eschlimann M. Malvé B. Goehringer F. Bensenane M. (2016). Characterization of hepatitis B virus surface antigen variability and impact on HBs antigen clearance under nucleos(t)ide analogue therapy. *J. Viral. Hepat.* 23 387–398. 10.1111/jvh.12498 26742490

[B136] Wai C. T. Fontana R. J. Polson J. Hussain M. Shakil A. P. Han S. H. (2005). Clinical outcome and virological characteristics of hepatitis B-related acute liver failure in the United States. *J. Viral. Hepat.* 12 192–198. 10.1111/j.1365-2893.2005.00581.x 15720535

[B137] Wang H. C. Wu H. C. Chen C. F. Fausto N. Lei H. Y. Su I. J. (2003). Different types of ground glass hepatocytes in chronic hepatitis B virus infection contain specific pre-S mutants that may induce endoplasmic reticulum stress. *Am. J. Pathol.* 163 2441–2449. 10.1016/s0002-9440(10)63599-7 14633616 PMC1892360

[B138] Wang H. Y. Chien M. H. Huang H. P. Chang H. C. Wu C. C. Chen P. J. (2010). Distinct hepatitis B virus dynamics in the immunotolerant and early immunoclearance phases. *J. Virol.* 84 3454–3463. 10.1128/jvi.02164-09 20089644 PMC2838120

[B139] Wang J. Yu Y. Li G. Shen C. Meng Z. Zheng J. (2018a). Relationship between serum HBV-RNA levels and intrahepatic viral as well as histologic activity markers in entecavir-treated patients. *J Hepatol* 68 16–24. 10.1016/j.jhep.2017.08.021 28870671

[B140] Wang J. Yu Y. Li G. Shen C. Li J. Chen S. (2018b). Natural history of serum HBV-RNA in chronic HBV infection. *J Viral Hepat* 25 1038–1047. 10.1111/jvh.12908 29633430

[B141] Wang J. Zhang P. Zeng J. Du P. Zheng X. Ye X. (2020). Occurrence of occult hepatitis B virus infection associated with envelope protein mutations according to anti-HBs carriage in blood donors. *Int. J. Infect. Dis.* 92 38–45. 10.1016/j.ijid.2019.12.026 31877352

[B142] Wang Y. Jiang L. Ji X. Yang B. Zhang Y. Fu X. D. (2013). Hepatitis B viral RNA directly mediates down-regulation of the tumor suppressor microRNA miR-15a/miR-16-1 in hepatocytes. *J. Biol. Chem.* 288 18484–18493. 10.1074/jbc.m113.458158 23649629 PMC3689990

[B143] Weber B. (2005). Genetic variability of the S gene of hepatitis B virus: clinical and diagnostic impact. *J. Clin. Virol.* 32 102–112. 10.1016/j.jcv.2004.10.008 15653412

[B144] Weber M. Bronsema V. Bartos H. Bosserhoff A. Bartenschlager R. Schaller H. (1994). Hepadnavirus P protein utilizes a tyrosine residue in the TP domain to prime reverse transcription. *J. Virol.* 68 2994–2999. 10.1128/jvi.68.5.2994-2999.1994 7512155 PMC236789

[B145] WHO (2020). *World’s Hepatitis Day.* Geneva: WHO

[B146] Will H. Reiser W. Weimer T. Pfaff E. Buscher M. Sprengel R. (1987). Replication strategy of human hepatitis B virus. *J. Virol.* 61 904–911. 10.1128/jvi.61.3.904-911.1987 3806799 PMC254036

[B147] Wu C. Zhang X. Tian Y. Song J. Yang D. Roggendorf M. (2010). Biological significance of amino acid substitutions in hepatitis B surface antigen (HBsAg) for glycosylation, secretion, antigenicity and immunogenicity of HBsAg and hepatitis B virus replication. *J. Gen. Virol.* 91(Pt. 2) 483–492. 10.1099/vir.0.012740-0 19812261

[B148] Wu H. L. Chen P. J. Tu S. J. Lin M. H. Lai M. Y. Chen D. S. (1991). Characterization and genetic analysis of alternatively spliced transcripts of hepatitis B virus in infected human liver tissues and transfected HepG2cells. *J. Virol.* 65 1680–1686. 10.1128/jvi.65.4.1680-1686.1991 1705988 PMC239971

[B149] Wu S. X. Chen W. N. Jing Z. T. Liu W. Lin X. J. Lin X. (2018). Hepatitis B spliced protein (HBSP) suppresses fas-mediated hepatocyte Apoptosis via activation of PI3K/Akt signaling. *J. Virol.* 92:e1273-18. 10.1128/JVI.01273-18 PMC6232459 30209179

[B150] Xia Y. Guo H. (2020). Hepatitis B virus cccDNA: formation, regulation and therapeutic potential. *Antiviral. Res.* 180:104824. 10.1016/j.antiviral.2020.104824 32450266 PMC7387223

[B151] Xu Z. Jensen G. Yen T. S. (1997). Activation of hepatitis B virus S promoter by the viral large surface protein via induction of stress in the endoplasmic reticulum. *J. Virol.* 71 7387–7392. 10.1128/jvi.71.10.7387-7392.1997 9311817 PMC192084

[B152] Yaginuma K. Nakamura I. Takada S. Koike K. (1993). A transcription initiation site for the hepatitis B virus X gene is directed by the promoter binding protein. *J. Virol.* 67 2559–2565. 10.1128/jvi.67.5.2559-2565.1993 8474161 PMC237576

[B153] Yan H. Zhong G. Xu G. He W. Jing Z. Gao Z. (2012). Sodium taurocholate cotransporting polypeptide is a functional receptor for human hepatitis B and D virus. *e*L*ife.* 13:e00049. 10.7554/eLife.00049 PMC3485615 23150796

[B154] Yang Y. C. Chen Y. H. Kao J. H. Ching C. Liu I. J. Wang C. C. (2020). Permanent inactivation of HBV genomes by CRISPR/Cas9-mediated non-cleavage base editing. *Mol. Ther. Nucleic Acids* 20 480–490. 10.1016/j.omtn.2020.03.005 32278307 PMC7150432

[B155] Yang P. Markowitz G. J. Wang X. F. (2014). The hepatitis B virus-associated tumor microenvironment in hepatocellular carcinoma. *Nat. Sci. Rev.* 1 396–412. 10.1093/nsr/nwu038 PMC4346158 25741453

[B156] Yen T. T. Yang A. Chiu W. T. Li T. N. Wang L. H. Wu Y. H. (2016). Hepatitis B virus PreS2-mutant large surface antigen activates store-operated calcium entry and promotes chromosome instability. *Oncotarget* 7 23346–23360. 10.18632/oncotarget.8109 26992221 PMC5029631

[B157] Zhang X. Zhang H. Ye L. (2006). Effects of hepatitis B virus X protein on the development of liver cancer. *J. Lab Clin. Med.* 147 58–66. 10.1016/j.lab.2005.10.003 16459163

[B158] Zhang Y. Li C. Zhang Y. Zhu H. Kang Y. Liu H. (2013). Comparative analysis of CpG islands among HBV genotypes. *PLoS One* 8:e56711. 10.1371/journal.pone.0056711 23451072 PMC3579858

[B159] Zhang Y. Mao R. Yan R. Cai D. Zhang Y. Zhu H. (2014). Transcription of hepatitis B virus covalently closed circular DNA is regulated by CpG methylation during chronic infection. *PLoS One* 9:e110442. 10.1371/journal.pone.0110442 25337821 PMC4206413

[B160] Zhao K. Liu A. Xia Y. (2020). Insights into hepatitis B virus DNA integration-55 years after virus discovery. *Innovation* 1:100034. 10.1016/j.xinn.2020.100034 PMC8454683 34557710

[B161] Zhao L. H. Liu X. Yan H. X. Li W. Y. Zeng X. Yang Y. (2016). Genomic and oncogenic preference of HBV integration in hepatocellular carcinoma [published correction appears in Nat Commun. 2016 Nov 08;7:13591. Lee, TP [corrected to Lee, TL]]. *Nat. Commun.* 7:12992. 27703150 10.1038/ncomms12992 PMC5059470

[B162] Zheng X. Weinberger K. M. Gehrke R. Isogawa M. Hilken G. Kemper T. (2004). Mutant hepatitis B virus surface antigens (HBsAg) are immunogenic but may have a changed specificity. *Virology* 329 454–464. 10.1016/j.virol.2004.08.033 15518823

[B163] Zhong H. Xibing G. Yaping D. Zheng W. Decai F. Xiaoye G. (2016). Interleukin-7 in patients with chronic hepatitis B may have effect on T follicular helper cells and specific cellular immunity. *Hepat Mon.* 16:e36068. 27822258 10.5812/hepatmon.36068 PMC5091030

[B164] Zhou Y. Holmes E. C. (2007). Bayesian estimates of the evolutionary rate and age of hepatitis B virus. *J. Mol. Evol.* 65 197–205. 10.1007/s00239-007-0054-1 17684696

[B165] Zhu A. Liao X. Li S. Zhao H. Chen L. Xu M. (2019). HBV cccDNA and its potential as a therapeutic target. *J. Clin. Transl. Hepatol.* 7 258–262. 31608218 10.14218/JCTH.2018.00054 PMC6783673

